# Food–Drug Interactions with Fruit Juices

**DOI:** 10.3390/foods10010033

**Published:** 2020-12-24

**Authors:** Zvonimir Petric, Irena Žuntar, Predrag Putnik, Danijela Bursać Kovačević

**Affiliations:** 1 Unit of Pharmacokinetics and Drug Metabolism, Department of Pharmacology at the Institute of Neuroscience and Physiology, Sahlgrenska Academy at the University of Gothenburg, 40530 Göteborg, Sweden; petric.zvonimir@gmail.com; 2 Faculty of Pharmacy and Biochemistry, University of Zagreb, Ante Kovačića 1, 10000 Zagreb, Croatia; 3 Department of Food Technology, University North, Trg dr. Žarka Dolinara 1, 48000 Koprivnica, Croatia; 4 Faculty of Food Technology and Biotechnology, University of Zagreb, Pierottijeva 6, 10000 Zagreb, Croatia; dbursac@pbf.hr

**Keywords:** fruit juice, interaction, drug, phytochemical, pharmacokinetics

## Abstract

Fruit juices contain a large number of phytochemicals that, in combination with certain drugs, can cause food–drug interactions that can be clinically significant and lead to adverse events. The mechanisms behind such interactions are in most cases related to phytochemical interference with the activity of cytochrome P450 metabolizing enzymes (CYPs) or drug transporters. Moreover, alterations in their activity can have a clinical relevance if systemic exposure to the drug is decreased or increased, meaning that the pharmacological drug effects are suboptimal, or the drug will cause toxicity. In general, the common pharmacokinetic parameters found to be altered in food–drug interactions regarding fruit juices are the area under the concentration–time curve, bioavailability, and maximum plasma concentration. In most cases, the results from the drug interaction studies with fruit juices provide only limited information due to the small number of subjects, which are also healthy volunteers. Moreover, drug interactions with fruit juices are challenging to predict due to the unknown amounts of the specific phytochemicals responsible for the interaction, as well as due to the inter-individual variability of drug metabolism, among others. Therefore, this work aims to raise awareness about possible pharmacological interactions with fruit juices.

## 1. Introduction

By definition, drug interactions (DIs) occur when the pharmacological effect of one drug is altered by the presence of another drug or xenobiotic, which includes herbal medicine, food or drink bioactive components, or any other chemical agents. DIs, if considered to be clinically significant, pose a risk for human health as they can have a direct effect on the therapeutic outcome and even cause life-threatening adverse drug reactions. Traditionally, mechanisms of DIs are classified as pharmacokinetic or pharmacodynamic, depending on the nature of the interactions. Pharmacokinetic DIs involve processes related to drug absorption, distribution, metabolism, and elimination, whereas pharmacodynamic DIs are those in which the drug effects are changed due to the presence of another drug or xenobiotic at its site of action [1]. In other words, depending on the nature of the DIs, different outcomes can be expected, such as decreased drug effectiveness with(out) increased drug toxicity or increased drug effectiveness with(out) increased drug toxicity. Hence, in the context of food–drug interactions, the clinical consequences can be the same, or expected, as in drug–drug interactions [2,3].

As a healthy lifestyle is nowadays becoming an imperative for many people, fruit juices recently came in the spotlight as a novel class of functional beverages, as they are promising carriers of biologically active compounds from many other food sources [4]. Namely, fruit juices, due to the recent advances in food (bio)technology, are now able to provide even more nutritional and health benefits to those seeking a well-balanced diet [5]. Given the fact that many bioactive compounds can be added to fruit juices, it is important to emphasize their clinical, pharmacological, and toxicological aspects regarding the potential of their involvement in DIs [2,3,6].

## 2. Absorption, Distribution, Metabolism and Elimination

Pharmacokinetics (PK) is a subdiscipline of pharmacology that quantitatively studies how a drug behaves in the body, i.e., what the body does to the drug. More specifically, the acronym ADME (absorption, distribution, metabolism and elimination) is often used to describe the PK processes of many drugs. PK principles can also be applied to patients (clinical pharmacokinetics) in order to provide safe and effective pharmacotherapy. To summarize, PK provides a quantitative relationship between a given dose and observed concentrations of a drug as a function of time, to provide optimization of dosage regimens. Pharmacodynamics (PD), on the other hand, is a subdiscipline of pharmacology, which studies the relationship between the drug concentration (at the site of the action, i.e., receptors) and the drug effects (response); i.e., what the drug does to the body. The pharmacological drug effect (or response) can be therapeutic and/or toxic, depending on the drug exposure. Hence, the PK/PD relationship can be viewed as an exposure–response relationship. Additionally, the relationship between the therapeutic and toxic dose of a drug is expressed by the therapeutic index, which tells about the relative safety of a drug or the narrowness of the therapeutic index, where a relatively small increase in the plasma drug concentration can lead to adverse effects and cause toxicity [7,8,9,10,11].

When the drug is orally taken, it must be absorbed, and this fraction is then carried into the hepatic portal system and liver before reaching the site of action in the unchanged state (systemic circulation). However, there is a possibility of drug metabolism (loss of a drug) along that path in gastrointestinal tissues and the liver. This loss of a drug is called the (presystemic) first-pass effect (metabolism). First-pass metabolism is an enzyme-catalyzed process, where the most common enzymes are of the cytochrome P450 (CYP) type. Besides, various isozymes, such as CYP3A4 (55% of all drugs) and CYP2D6 (30% of all drugs), among many others, are involved in first-pass drug metabolism and show inter-individual variability; i.e., differing enzymatic activity. This activity is of particular interest in determining many DIs, as drugs, bioactive compounds from food (fruit juices), and other xenobiotics, when co-administered, can inhibit or induce CYP activity (CYP inhibitors and CYP inducers), consequently affecting the pharmacodynamic outcome [7,8,9,10,11]. One of the PK parameters often mentioned in this paper is the area under the drug concentration–time curve (AUC), which is used to calculate the bioavailability (F) of a drug. The AUC shows how much of a drug is in the body or describes the total systemic exposure to the drug. Furthermore, F is the fraction (or percentage) of the absorbed drug that will be available at the site of action. In other words, F tells us about the rate and extent of drug absorption. F is a PK parameter that also indicates the extent of the systemic exposure to the drug. Additionally, for the intravenous route of drug administration (i.v.), F is by definition 100% (F = 1). The AUC of the orally administered drug relative to the AUC of the i.v. route gives us the possibility to calculate the absolute oral bioavailability of the drug (Foral). Foral can also be viewed as the product of the fractions of a drug dose that escapes metabolism by the gut (FG) and liver (FH); i.e., Foral = Fabs × FG × FH, where Fabs is the fraction of a dose that is absorbed intact across the enterocytes [7,8,9,10,11].

Many factors can influence the rate of drug absorption, such as P-glycoprotein (drug transporter), and many factors can influence the extent of the drug absorption, such as the first-pass metabolism. Another PK parameter that is mentioned in the paper is the elimination half-life (t_1/2_), which can be obtained from the concentration–time profile of a drug, which gives us information of the time needed to eliminate half of the drug in the body (after drug distribution is over); simply put, it describes the decay of the drug. Moreover, the half-life determines the duration of drug-action for many medications. Half-life is dependent on drug clearance (CL), as CL relates to the rate of drug elimination and drug systemic exposure. The higher the CL, the lower the systemic exposure (AUC). In addition, C_max_ is the peak concentration, or maximum plasma concentration of the drug, which can be easily obtained from the AUC [7,8,9,10,11]. Finally, the number of possible DIs is without a limit, but as a general statement, it can be said that the most pronounced DIs will be those that are affecting oral bioavailability and clearance of drugs, particularly by affecting drug metabolism. DIs are one of the important sources of drug toxicity, as well as responsible for the variable therapeutic responses among individuals [7,8,9,10,11].

## 3. Importance of Cytochrome P450 Enzymes and Drug Transporters in Drug Interactions

Humans have substrate-specific enzymes (e.g., CYP enzymes/CYP system) that are found in different tissues (e.g., liver, intestine, lungs, etc.), which makes the entire DI concept even more complex, and especially so if the polymorphisms in cytochrome P-450 genes are taken into count. This means that CYP activity shows interindividual differences that is reflected in the drug PK and can make a direct impact on DIs. Moreover, due to various drugs and xenobiotics, which can act as CYP inhibitors or inducers, there also can be intra-individual differences in drug response and toxicity. Additionally, intrinsic factors (such as age, sex, disease, etc.) combined with extrinsic factors (diet, lifestyle, smoking, etc.) further contribute to a variable drug response, also making the prediction of a DI even more challenging for an individual patient.

The CYP system plays an important role in the metabolism/biotransformation of drugs and xenobiotics (as for detoxification), and they are therefore one of the most important factors contributing to DIs, including food–drug interactions [12]. In short, the CYP system is a large family of hemoproteins that catalyze a wide range of reactions (hydroxylation, epoxidation, oxygenation, dealkylation, isomerization, desaturation, reduction, etc.). The common CYPs that are clinically significant for DIs include CYP3A4, CYP2D6, CYP2C9, CYP2C19, CYP2B6, CYP2E1 and CYP1A2 [13]. Hence, any of these chemical reactions in the body can be influenced by food components, including the ones from fruit juices, which consequently can induce, or inhibit, one or more CYP enzymes, with repercussions on drug therapeutic exposure.

Besides the CYP system, drug transporters also have an important role in drug and xenobiotic pharmacokinetics. In general, transporters can be categorized as uptake and efflux transporters, and can be divided into two superfamilies (with more than 500 members), namely, the ATP-binding cassette (ABC) family and the solute carrier (SLC) family. Some of them are ubiquitously expressed, while some are found in tissues such as the liver, brain, small intestine and kidney. Some of the transporters, often being clinically relevant to DIs, include P-glycoprotein (P-gp), also known as multidrug resistance protein 1 (MDR1), breast cancer resistance protein (BCRP), organic anion transporter (OAT1, and OAT3), organic cation transporter (OCT2) and organic anion transporting polypeptide (OATP1B1 and OATP1B3) [14,15]. It is important to mention that drug transporters can also have functional genetic polymorphisms just like CYP system [16].

**Figure 1:**
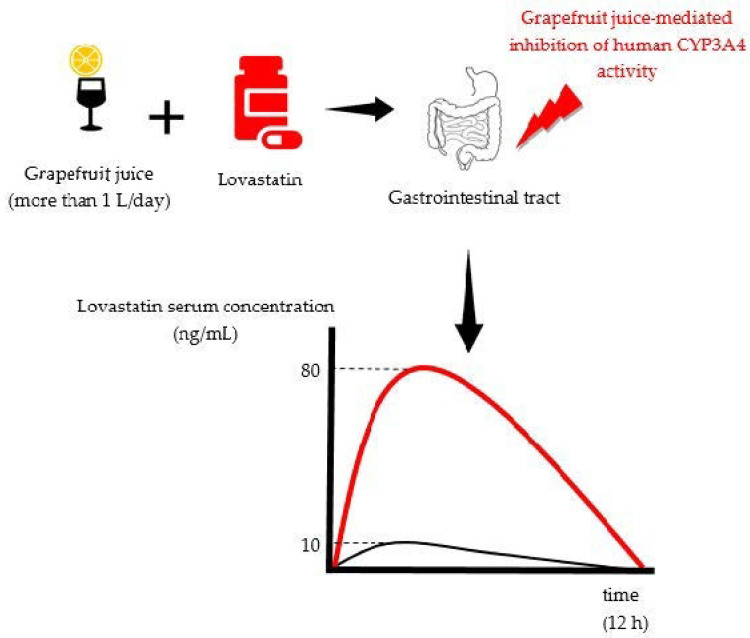
Hypothetical PK profile of lovastatin taken with water (black curve) and with grapefruit juice (red curve). The noticeable increase in the AUC (red curve) implicates an increase in systemic drug exposure, leading to the development of adverse effects; i.e., myopathy and rhabdomyolysis (the C_max_ of the lovastatin taken with water was 7 ± 2.5 ng/mL, while for the grapefruit juice it was 82.4 ± 39.6 ng/mL. The FDA label of lovastatin tablet (40 mg) states that, at the steady-state, the C_max_ of lovastatin should be 7.8 ng/mL) [29,32,33].

Altogether, the clinical relevance of the CYP system and drug transporters is recognized in drug–drug interactions, but also in food–drug interactions, because different xenobiotics, including those from fruit juices, e.g., grapefruit juice, can influence their activity and thus have an impact on the systemic drug exposure; i.e., efficacy and toxicity (Figure 1) [17,18,19]. More importantly, the reader should be aware that drug interactions shown in vitro does not necessarily mean they are also occurring in vivo. Moreover, even if they do occur in vivo (animals), their clinical relevance for humans still could be insignificant, and vice versa. This can be seen in an example of a DI between pomegranate juice and flurbiprofen (a non-steroidal anti-inflammatory agent) [20], which shows discrepancies between in vitro and clinical studies [21].

## 4. Fruit Juices

Fruits (and vegetables) contain structurally diverse bioactive compounds, such as flavonoids (flavonols, flavones, flavanones, flavanols, anthocyanidins and isoflavones), phenolic acids (hydroxybenzoic acids and hydroxycinnamic acids), carotenoids (β-carotene, lycopene, lutein, zeaxanthin, flavoxanthin, canthaxanthin, capsanthin, capsorubin and β-cryptoxanthin), vitamins (vitamin C–ascorbic acid and vitamin E–tocopherols) and phytoestrogens (isoflavonoids, stilbenes, lignans, coumestans and glucosinolates). Furthermore, it is estimated that almost 5000 phytochemicals have been found in fruits (and vegetables), but a large proportion remains to be discovered. The bioactive compounds in fruits are also greatly variable in amounts due to various parameters, such as the stage of ripeness, cultivar/variety, agricultural practices, environment, harvest and postharvest procedures, processing, storage, etc. [22]. Since most of the fresh fruit is processed, the food industry is increasingly applying new non-thermal processing technologies to preserve the original nutritional and sensory quality of the product, with an emphasis on the preservation of bioactive compounds to the greatest extent [5,23]. Therefore, it can be concluded that fruits (and vegetables) contain a complex combination of constituents, so predicting and determining their interactions with drugs, and the clinical relevance, is very challenging. At this point, it is important to define what is a fruit juice. Even though there are various definitions, the one according to the UK Fruit Juice and Fruit Nectars Regulations seems reasonable, as it gives a broader view of the definition:

“Fruit juice is the fermentable but unfermented product obtained from the edible part of the fruit which is sound, ripe and fresh or preserved by chilling or freezing of one or more kinds mixed having the characteristic color, flavor, and taste typical of the juice of the fruit from which it comes.”

However, the specifications and regulations depend on the different types of fruit juices, such as fruit juice from concentrate, concentrated fruit juice, water-extracted fruit juice and dehydrated and powdered fruit juice. Within the EU, fruit juices are regulated by Council Directive 2001/112/EC [24]. Smoothies, on the other hand, do not have a legal definition, but they can contain fruit purees, fruit juice or crushed parts of the fruit. Fruit juices, compared to other foods, contain more beneficial nutrients, especially calcium, iron, vitamin A, thiamin (vitamin B1), riboflavin (vitamin B2) and ascorbic acid (vitamin C), and many other antioxidants, such as tocopherols (vitamin E), beta-carotene, flavonoids, fibers and other minerals [25,26]. It should be kept in mind that fruit juices can also be carriers for many bioactive compounds, which are additionally added to make a novel class of beverages, i.e., functional fruit juices. Hence, concerns regarding interactions between fruit juices and drugs should not sound surprising, especially if we are witnessing clinically significant DIs and unwanted pharmacological outcomes linked to their concomitant consumption [27].

## 5. Drug Interactions with Common Fruit Juices

The most extensively described DIs with fruit juices are those with grapefruit juice (and grapefruit pulp), for which it is reported to have more than 40 DIs in humans [28]. Therefore, grapefruit juice that was consumed with the medicine lovastatin was used as a representative example for illustration of a DI, shown in Figure 1. The figure shows the CYP3A4 inhibition by the grapefruit juice, leading to development of adverse effects; i.e., myopathy and rhabdomyolysis. Additionally, a human PK study confirmed that C_max_ and the AUC of lovastatin, when taken with grapefruit juice, were increased about 12-fold and 15-fold, respectively [29,30,31].

The discovery of the grapefruit interaction with drugs was an unexpected result of an interaction study with a focus on ethanol and felodipine (a calcium-channel antagonist) that targeted masking the taste of ethanol with grapefruit juice. The results showed an increase in felodipine bioavailability and C_max_, as a consequence of irreversible degradation of an intestinal CYP3A by the grapefruit compounds [30,31]. Moreover, grapefruit juice was found to have an impact on the efflux transport via P-glycoprotein in vitro, but also the influx transport via organic anion-transporting polypeptides—OATPs, esterases and sulfotransferases—although the clinical relevance of grapefruit juice on these drug transporters and enzymes was never determined [34]. Other drug classes that are known to have a clinically relevant interaction with grapefruit juice include 3-hydroxy-3-methylglutaryl coenzyme A (HMG-CoA) reductase inhibitors (simvastatin), immunosuppressives (cyclosporine), antiarrhythmics (amiodarone) and anticonvulsants (carbamazepine). However, in some other cases with other drugs, even if the pharmacokinetic parameters were altered by the interaction with grapefruit, the clinical significance was still considered insignificant [34,35]. In addition, it should be kept in mind that, due to the variable amounts of phytochemicals in juices, the results between the different pharmacokinetic studies of the same fruit juices cannot be compared, but the outcome can still be intuitively predicted.

The literature suggests that the specific components in grapefruit juice related to DIs could involve furanocoumarins, namely, bergamottin and 6′7′-dihydroxbergamottin [36], but also naringin, which can inhibit OATP1A2 activity [21]. It is speculated that even micromolar concentrations of naringin were responsible for a DI with fexofenadine, resulting in a decrease of its bioavailability [37], which in this case could be clinically significant but was not confirmed by other authors.

There are some indications that grapefruit juice can reduce the levels of CYP3A4 by 47% only four hours after consumption, and these effects are persistent in the intestinal and liver cells at least 24 h after ingestion, meaning that grapefruit juice, even without co-administration with the drug, can lead to a DI or alter the metabolism of any CYP3A4 substrate for longer periods of time after consumption [38]. Proof for that can be seen in the example of a DI with tacrolimus (immunosuppressant), where a delayed increase in systemic exposure to tacrolimus, e.g., from 4.7 ng/mL to 47.4 ng/mL, happened one week after the last grapefruit juice intake (250 mL, 4 times a day for 3 days). The patient developed severe headache and nausea, luckily without nephrotoxicity [28,39]. Additionally, there is a case report of statin-associated rhabdomyolysis triggered by grapefruit consumption. Rhabdomyolysis is a rare but very serious adverse effect associated with statin therapy, which could cause kidney failure and death. In this case, the metabolism of simvastatin (CYP3A4 substrate) was altered due to CYP3A4 inactivation by the grapefruit, resulting in toxic systemic exposure to simvastatin [40]. What could be expected from this data is that other statins, such as atorvastatin, which are CYP3A4 substrates, will have the same unwanted clinical outcomes. However, statins that are not substrates for the same CYP, such as rosuvastatin, are not expected to have this DI.

Altogether, it is clear that grapefruit juice interactions with orally given drugs cannot be generalized, and clinical relevance cannot be precisely determined. Hence, it is better to avoid grapefruit consumption with drugs that are substrates for an intestinal CYP3A4 and/or P-gp, especially in the cases when a drug has a narrow therapeutic index and a poor oral bioavailability due to the high pre-systemic (first-pass) metabolism mediated via CYP3A4, because even a single consumption of grapefruit juice can lead, in some cases, to drug toxicity. Additionally, the concentration/amount of the grapefruit extracts and as well interindividual variability of intestinal CYP3A4 activity among humans also contribute to the severity of the unwanted outcome [35,41].

Besides grapefruit, other citrus juices, such as orange, lemon, pomelo, and lime, were also reported to cause DIs in some clinical studies. As previous results were contradictory, or the clinical relevance could not be determined, a meta-analysis from Sridharan et al. provides a good summary of the DIs with selected citrus juices and cyclosporine (substrate for CYP3A4). Cyclosporine is an immunosuppressive drug with a narrow therapeutic index, with variable pharmacokinetics from person to person. Pooled results showed that no significant changes in the AUC and C_max_ of cyclosporine were observed with orange juice as compared to the controls, while pomelo juice, on the other hand, increased the AUC and C_max_ and decreased the elimination t_1/2_ of cyclosporine [42]. Hence, orange juice did not have any significant interaction with cyclosporine. Interestingly, an earlier review from different authors [43], regarding orange juice, reported quite the opposite trend. Furthermore, orange juice does not contain bergamottin derivatives that are present in grapefruit juice, which may answer why orange juice did not have any significant interaction with cyclosporine. Pharmacologically, it would be expected that DIs with citrus juices (except orange juice) would have a more significant meaning in the case of liver disease and the concomitant use of other drugs (often seen in elderly patients) that share the same enzymatic pathway.

Orange juice was in one study found to decrease the oral absorption of alendronate (bisphosphonate agent for the treatment of osteoporosis) by approx. 60% compared to water [44]. This is very important, as alendronate, if taken on an empty stomach (after an overnight fast), 2 h before any meal (breakfast), has by default a very poor absolute oral bioavailability—approx. only 0.75% of the total dose [45]. Hence, taking orange juice with alendronate should be avoided.

Seville (sour) orange juice was found to have the same mechanism of CYP3A4 inactivation as grapefruit juice, and thus interacts with felodipine [46]. However, Seville orange juice did not alter the bioavailability of cyclosporine despite the CYP3A4 activity being significantly reduced, in contrast to grapefruit juice [47]. Therefore, it seems that grapefruit juice, besides the inactivation of intestinal CYP3A4, alters some other enzymes or transporters.

Seville orange and lime juices were in one clinical study inspected for a DI with sildenafil, a drug mostly known as Viagra^®^, which is an agent that improves penile erectile function [48]. Sildenafil has an extensive first-pass metabolism, which results in a relatively low absolute oral bioavailability (F = 40%). Namely, for healthy subjects that received sildenafil (single dose of 50 mg) for three consecutive days and drank 250 mL of juice (or water) just before the drug was taken, the results showed that Seville orange juice increased the AUC and C_max_ of sildenafil by 44%. Although Seville orange juice is considered a moderate CYP3A4 inhibitor, this interaction did not have any adverse effects. The increase in systemic exposure is believed to be due to the intestinal inhibition of CYP3A4 and P-gp. On the other hand, lemon juice did not show any effects on the PK parameters of sildenafil.

Pomelo fruit juice (250 mL) was also inspected for a DI with sildenafil in another clinical study [49]. Surprisingly, pomelo juice decreased the systemic exposure to sildenafil. Namely, the bioavailability of sildenafil with pomelo juice was 60% lower compared with water. This is explained by a possibility that there was some sort of physicochemical interaction with one of the phytochemicals from pomelo juice, or an interaction with some drug transporter. However, the clinical significance of this interaction was not established, but the authors suggested avoiding taking pomelo juice with sildenafil.

Based on the previous information, it can be predicted that sildenafil, as a substrate for CYP3A4, will also have a DI with grapefruit juice, resulting in an increase in the systemic exposure of sildenafil. This was also confirmed in humans, but the adverse effects were not noticed [50]. However, it seems reasonable to avoid this combination. Namely, there are three independent case reports of DI-induced priapism (a persistent and painful penile erection, which is an emergency due to the risk of impotency) due to the concomitant use of pomegranate fruit juice (a high content of flavonoids) and sildenafil [51]. Although evidence of this interaction is based on circumstances, it is still a good example of how DIs with fruit juices are clinically significant, and adverse effects that are generally rare, such as priapism, are believed to be precipitated by a fruit juice. The proposed mechanism behind this interaction is due to CYP3A4 inhibition by phytochemicals from pomegranate juice.

Dresser et al. examined the effect of apple and orange juice on OATP uptake transporters [52]. Namely, in a human volunteer study, subjects were given two fexofenadine tablets (60 mg) (antihistaminic, sold by the trade name Allegra^®^) with 300 mL of fruit juice, up to a total volume of 1.2 L. The results of the PK study showed that both juices significantly decreased the fexofenadine concentrations in plasma compared with water (AUC (fexofenadine) apple juice = 434 ± 53; AUC (fexofenadine) orange juice = 494 ± 16; and AUC (fexofenadine) water = 1616 ± 120, *p* < 0.001). Among the tested individuals, the extent of the decreased fexofenadine concentrations was variable, or individuals with the highest fexofenadine AUC with water had the greatest decrease with the juices. Fexofenadine is a substrate of P-gp, but as well as of OATP uptake transporters, so Dresser et al. suggest the necessity to determine the individual contribution of each transporter in this case.

Apple juice was also found to have a DI with atenolol, which is antihypertensive drug [53]. In a human volunteer PK study, atenolol (50 mg) was taken with apple juice (600 mL and 1200 mL) and it was shown that atenolol systemic exposure was inversely proportional to the amount of consumed apple juice as compared to water (AUC (atenolol) 600 mL apple juice = 885.3; AUC (atenolol) 1200 mL apple juice = 389.7; and AUC (atenolol) water = 2110). In other words, the AUC of atenolol was decreased by 82% after the ingestion of 1200 mL apple juice, but there were no observed changes in the pharmacodynamic outcome even though there is an evident dose–response relationship. This DI is believed to be due to inhibition of intestinal drug transporter OATP2B1, but it is also believed that the higher acidity in the gastrointestinal tract (a large amount of apple juice) could also have an impact on atenolol absorption.

Other drugs reported as having a decreased systemic exposure with apple juice were montelukast (an anti-asthma agent) and aliskiren (an antihypertensive agent), which are also substrates for uptake transporters [54]. In addition, decreasing the systemic exposure of atenolol and montelukast was also noticed with orange juice [55,56]. However, to our knowledge, there are no documented cases of a DI (from clinical practice) with apple and orange juice to date. One exception could be in the case of calcium-fortified orange juice, where, due to chelation of the drugs with calcium, alterations in the C_max_ and AUC of the fluoroquinolones (antibiotics) could decrease the antibiotic effects, leading to antibiotic resistance [57,58]. The latter statement could be applied to all calcium-fortified fruit juices.

It is worth mentioning that orange juice was found to increase the systemic exposure to aluminum [59] and iron [60] by enhancing their absorption. In the case of aluminum, there is, in theory, a risk of aluminum toxicity in a patient with renal disease, as it was found that urinary excretion of aluminum increased 10 times when it was taken with orange juice.

Fruits, such as oranges, bananas and prunes, along with their fruit juices, or in common combinations with vegetable juices such as carrot juice and tomato juice, contain very high amounts of potassium, which in combination with potassium-sparing drugs, such as angiotensin-converting enzyme inhibitors (ramipril), diuretics (spironolactone, triamterene) and especially in patients with kidney disease (hemodialysis patients) and with hypoaldosteronism, could lead to life-threatening hyperkalemia, so it is advisable to always seek advice from a pharmacist prior to consumption [61,62,63]. Prune juice is best known for its laxative effect, so it is often sold as an over-the-counter (OTC) dietary supplement in many pharmacies and specialized stores, where consumers/patients might be unaware of the possible side effects [64,65].

Plum and avocado juices were found to contain higher amounts of biogenic amine, tyramine, as compared to other fruit sources [66]. In theory, monoamine oxidase inhibitors (MAOIs) can inhibit tyramine degradation, which leads to its increased systemic exposure with a consequence of developing a hypertensive crisis [67]. However, over the last decades, the use of MAOIs has decreased and documented cases of their toxicity (due to the tyramine overexposure) are very rare, but the caution is still advised, as even 8 mg of tyramine was historically known to cause hypertension with some MAOIs [68,69].

Cranberry fruit juice is nowadays a very popular prophylactic treatment for the infections of the urinary tract. There have been some safety concerns regarding the cranberry fruit juice intake and using warfarin—an anticoagulant—which is known for its narrow therapeutic index and life-threatening side effects in case of increased systemic exposure [70,71]. The main reason for concern was a clinical case report that reported a change in the INR (International Normalized Ratio), a biochemical marker of warfarin’s antithrombotic effects, related to cranberry juice intake. INR was in some patients decreased, meaning that the blood coagulates too easily, so the risk for developing blood clots rises [72], or it was increased, meaning that there is increased risk of bleeding [73]. However, in a randomized, double-blind clinical study, this interaction was not confirmed [74]. Ansell et al. commented that cranberry juice was not the one to blame for the results, as the reported cases were not convincing beyond doubt. Namely, patients had a variety of illnesses, the amount of ingested cranberry juice was not known in some cases, as well as the other dietary components, nor was the patients’ compliance known, but most importantly, the pharmacogenetic profiling was not done either [75]. Although, it is well-established that polymorphisms of CYP2C9 and vitamin K epoxide reductase (VKORC) affect the INR values and clinical outcome of pharmacotherapy with warfarin. Detailed discussion of this interaction was provided by Zikria et al. [76]. In the literature, there are other described cases of other fruit juices that have in vitro effects on drug-metabolizing enzymes or transporters; however, their clinical significance was never firmly confirmed.

Tangerine fruit juice was reported to upregulate CYP3A4 activity and inhibit P-glycoprotein due to the high content of flavonoid tangeretin [77,78]. Interestingly, some older data reported the opposite effect—tangerine (tangeretin) inhibits CYP3A4 and CYP1A2 in human liver microsomes [79].

Black mulberry was reported to inhibit CYP3A (and OATP-B), but also was wild grape [80]. Black mulberry is traditionally promoted as a fruit with a high content of iron, so it has a beneficial effect on the treatment of anemia. In addition, some studies showed that black mulberry has a beneficial effect on cholesterol levels, liver tissue (hepatoprotective effect) and even anti-obesity potential [81]. In theory, large amounts of black mulberry fruit juice could interact with CYP3A4 substrates, leading to an increase in systemic exposure of such drugs, but clinical relevance was never determined in humans.

Mango stem-bark was found to inhibit CYP1A1, CYP1A2, CYP3A1, CYP2C6, CYP2E1 and P-glycoprotein [82], while grape, due to the resveratrol, was implied in inhibition of CYP1A1/1A2 isoforms and CYP2E1, which are needed for the activation of procarcinogens (polycyclic arylamines, polyaromatic hydrocarbons, aflatoxin B1 and *N*-nitrosamines, respectively). Hence, besides having protective role as antioxidants, it is suggested that their fruit juices could alter the CYP3A4 activity, which is an important biotransformation pathway for many drugs [83]. The same was reported for papaya [84] and black raspberry; however, a recent study with taxane agents (anticancer agents) did not confirm altered CYP3A4 activity [85].

Tropical fruit juices, such as pineapple, papaya, litchi, kiwi, starfruit and passion fruit, were implicated in DIs in vitro due to their inhibitory effects on CYP2C9 or CYP3A4. Pineapple fruit juice, due to a high bromelain content was found to have the most pronounced inhibitory properties on CYP2C9, compared to other fruit juices. The effect of the inhibition was proportionally dependent on the increase in the amount of pineapple juice. Moreover, starfruit juice was found to be a very potent inhibitor of CYP3A4 compared to grapefruit juice. Namely, an assay of midazolam 1-hydroxylase activity of human CYP3A showed that residual activity of midazolam 1-hydroxylase (%) with starfruit juice was only 0.1 ± 0.0, as compared to the 14.7 ± 0.5 for grapefruit juice. It would be interesting to study those inhibitory activities in vivo to determine the clinical relevance of tropical fruit juices on DI.

The next interesting question to address is, could fruit juices be exploited for enhancing the positive pharmacotherapy outcome in some cases? The answer is yes [86].

Grapefruit juice was shown to be a drug-sparing agent (an agent that decreases the therapeutic dose of another drug) in a case of concomitant use with cyclosporine. In other words, patients could avoid the dose-related side effects of cyclosporine [27,87]. In another study, grapefruit enhanced oral bioavailability of artemether, which is antimalarial agent with generally high presystemic metabolism via CYP3A4, indicating more effective treatments of malaria [88]. In other words, grapefruit juice could be useful in maintaining the effectiveness and efficacy of some drugs.

Lime juice and artemisinin combination therapy (antimalarial agents) were given in one study to children (61 males and 50 females) with acute uncomplicated malaria [89]. It was observed that the artemisinin with lime juice caused more rapid clearance of parasites; also, the lime juice is believed to prevent resistance. The proposed explanation for this phenomenon is high amounts of vitamin C and flavonoids present in lime juice, which, besides its low pH, contributed to antioxidant activity. Similar results were shown recently on murine models, but with a lemon decoction resulting in a suppression of parasites by 39% and rapid early parasite clearance, as compared to the controls. However, there is a need to determine the exact effects of lime and lemon juice as supplement treatments in malaria by doing further investigations.

Blueberry juice, prepared from fresh blueberries, was given to 201 children that were receiving etanercept as a treatment for their juvenile idiopathic arthritis [90]. The study showed that the blueberry juice treatment combined with etanercept improved the symptoms of the disease, but as well decreased the side-effects of etanercept.

Regarding the previously mentioned increase of iron absorption with orange juice, it seems that it has a beneficial effect and such a combination could contribute to a better response to iron-deficiency anemia, which is a common problem, especially in children, but also in adults [91]. As DIs precipitated by fruit juices can sometimes be predicted, Table 1 summarizes previously described examples of potentially relevant drug interactions that should be considered in everyday clinical practice.

## 6. Conclusions

Fruit juices contain a large number of phytochemicals, but there are not enough clinical studies to evaluate their roles in drug interactions, even though the literature provides evidence that some fruit juices can impact drug disposition and thus interact with drugs. Predicting drug interactions potentiated by fruit juices is challenging due to the unknown number of phytochemicals present in the fruit juice, the unknown doses, and the individual differences among individuals who consume them concomitantly with their medications. Hence, the pharmacodynamic outcome cannot be generalized.

Although many drug interactions with fruit juices are not considered clinically relevant, there still are some that deserve our attention. For the people who prefer avoiding any potential drug interaction precipitated by fruit juices, the best advice is to take the medication with water.

In conclusion, a better understanding of the mechanisms behind drug interactions with fruit juices, and further investigations, are still needed to decrease the adverse drug reactions associated with fruit juice consumption. Patients should always ask specialized healthcare professionals about any concerns regarding their medication and fruit juice interactions, in order to decrease the likelihood of any unwanted effects or unsuccessful pharmacological treatments.

## Figures and Tables

**Table 1:** Examples of potentially relevant drug interactions precipitated by fruit juices in humans.

Fruit Juice Type	Examples of Drugs	Suggested Mechanism of an Interaction	Reference
Grapefruit	CYP3A4 substrates HMG-CoA reductase inhibitors (simvastatin, lovastatin) Immunosuppressives (cyclosporine) Antiarrhythmics (amiodarone) Anticonvulsants (carbamazepine)	via CYP3A4, or/and P-gp	[34,35]
	PDE5 inhibitor (sildenafil)		[50,51]
	Antimalarial agent (artemether)		[88]
Orange	Bisphosphonates (alendronate)	physicochemical interaction	[45]
	Antihistamines (fexofenadine)	OATP transporters, or/and P-gp	[52]
	Beta-blocker (atenolol)		[55]
	Anti-asthmatic agent (montelukast)		[56]
Calcium-fortified orange juice	Aluminum and iron supplements	physicochemical interaction	[59,60]
	Antibiotics (fluoroquinolones)		[57,58]
Seville orange	PDE5 inhibitor (sildenafil)	via CYP3A4	[48]
Pomegranate	PDE5 inhibitor (sildenafil)	via CYP3A4	[50,51]
Pomelo	PDE5 inhibitor (sildenafil)	physicochemical interaction	[49]
	Immunosuppressives (cyclosporine)	P-gp	[42,43]
Apple	Antihistamines (fexofenadine)	OATP transporters, or/and P-gp	[52]
	Beta-blocker (atenolol)		[53]
	Anti-asthmatic agent (montelukast)		[54]
	Antihypertensive agent (aliskiren)		[54]
	Antihistamines (fexofenadine)		[52]
Blueberry	TNF-α inhibitor (etanercept)	Beneficial interaction suggested to be due to anti-oxidant/anti-inflammatory properties of blueberries	[90]

HMG-CoA reductase = 3-hydroxy-3-methyl-glutaryl-coenzyme A reductase; PDE5 = phosphodiesterase type 5; OATP = Organic Anion Transporting Polypeptides; P-gp = P-glycoprotein.
